# Infektionen mit den humanpathogenen Mykoplasmenspezies *M. genitalium* und *M. pneumoniae*: Aktuelle epidemiologische Aspekte

**DOI:** 10.1007/s00103-025-04052-2

**Published:** 2025-05-12

**Authors:** Roger Dumke

**Affiliations:** TU Dresden, Institut für Medizinische Mikrobiologie und Virologie, Konsiliarlabor des Robert Koch-Instituts für Mykoplasmen, Fetscherstraße 74, 01307 Dresden, Deutschland

**Keywords:** Sexuell übertragbare Infektion, Ambulant erworbene Pneumonie, Epidemiologie, Resistenz, Typisierung, Sexually transmitted infection, Community-acquired infection, Epidemiology, Resistance, Typing

## Abstract

Für die beiden humanpathogenen Mykoplasmenspezies *Mycoplasma pneumoniae* und *M. genitalium* ergeben sich derzeit unterschiedliche Herausforderungen für die klinische Praxis.

*M. pneumoniae *ist eine häufige Ursache von ambulant erworbenen respiratorischen Infektionen, die zu asymptomatischen Verläufen, aber auch zu schweren atypischen Pneumonien führen können. Hinzu kommen extrapulmonale Komplikationen. Seit Dezember 2023 werden in Sachsen, wo eine Meldepflicht besteht, Prävalenzen gemessen, die die Zahlen aus den vergangenen Jahren um ein Vielfaches übersteigen und auf die übrigen Bundesländer grundsätzlich übertragbar sind. Die Ursache dieser Entwicklung dürfte in einem reduzierten Kontakt mit dem Erreger während der COVID-19-Pandemie verbunden mit einer Verringerung der spezifischen Immunität in der Bevölkerung liegen. Obwohl es keine gesicherten Daten zu einem Anstieg der Rate schwerer Fälle gibt, sind Diagnostik und Therapie dieser Situation anzupassen. Die Resistenzrate in Deutschland gegenüber den vorrangig eingesetzten Makroliden bleibt mit ca. 3 % günstig.

*M. genitalium *wird sexuell übertragen und ist nur in einem Teil der beim Mann auftretenden Urethritisfälle ursächlich relevant. Der begrenzten epidemiologischen Bedeutung steht die zunehmende Resistenz gegenüber den in den Leitlinien empfohlenen Antibiotika Azithromycin und Moxifloxacin gegenüber, die in den im Konsiliarlabor in den vergangenen Jahren untersuchten Proben bei 69 % bzw. 25 % liegt. In Risikogruppen (z. B. bei Männern, die Sex mit Männern haben), in denen *M. genitalium* relativ häufig nachzuweisen ist, muss mit noch höheren Raten gerechnet werden. Ein Therapieerfolg ist unter diesen Bedingungen nur mit einem resistenzgeleiteten Behandlungskonzept zu erwarten.

Die vorliegende Übersicht fasst den momentanen Kenntnisstand zu beiden Erregern zusammen.

## Abstract (en)

Two human pathogenic *mycoplasma* species, *Mycoplasma pneumoniae* and *M. genitalium *currently pose challenges for the clinical practice.

*M. pneumoniae *is a common cause of community-acquired respiratory infections, which can lead to asymptomatic courses as well as severe atypical pneumonia. Extrapulmonary manifestations also occur. Since December 2023, a strong increase of reported cases has been registered in the federal state of Saxony, where reporting is mandatory. These numbers far exceed those from the previous ten years and are assumed to generally translate to the other federal states. The reason for this development is likely the reduced contact with the pathogen during the COVID-19 pandemic combined with a waning immunity in the population. Although there are no reliable data on an increase in the rate of severe cases, diagnostics and treatment must be adapted. The resistance rate to macrolides (which are primarily used in Germany) remains favorable at approximately 3%.

*M. genitalium *is a sexually transmitted bacterium and is only causally relevant in a portion of male urethritis cases. While its epidemiological significance is limited, resistance to the guideline-recommended antibiotics azithromycin and moxifloxacin is increasing, reaching 69% and 25%, respectively, in samples tested in the German consulting laboratory in recent years. Even higher rates are to be expected in high-risk groups (e.g., men who have sex with men), where *M. genitalium* is relatively frequent. Under these conditions, therapy can only be successful with a resistance-guided treatment concept.

This review summarizes the current state of knowledge on both pathogens.

## Einleitung

Mykoplasmen sind als Krankheitserreger bei Pflanzen, Tieren und dem Menschen zu finden. In der evolutionären Interaktion mit den jeweiligen Wirten haben diese Bakterien wesentliche Teile ihrer genetischen Ausstattung verloren. Mit Genomgrößen zwischen 0,5 und 1,3 Megabasenpaaren (Mbp.) sind sie gegenwärtig die kleinsten bekannten Mikroorganismen, die sich selbst replizieren können. Mit der Genomreduktion ist eine Limitierung der Stoffwechselleistungen verbunden. So fehlt Mykoplasmen u. a. der Tricarbonsäurezyklus, die Aminosäure‑, Nukleotid- und Fettsäuresynthese sind ebenfalls nicht vorhanden oder stark eingeschränkt. Sie sind zu ihrer Vermehrung deshalb auf Substanzen angewiesen, die von den infizierten Wirtsorganismen zur Verfügung gestellt werden. Mykoplasmen sind somit hoch angepasste, parasitär lebende Bakterien. Obwohl überwiegend kultivierbar, stellen sie hohe Ansprüche an die angebotenen Nährmedien und weisen relativ lange Generationszeiten auf. So sind Sterole für das Wachstum notwendig; ein für die Vermehrung von Bakterien unübliches Additiv. Aus der Vermehrungskinetik resultieren nach der Infektion des Wirtes relativ lange Inkubationszeiten. Neben dem begrenzten Stoffwechsel ist das Fehlen einer bakteriellen Zellwand die gravierendste Folge der Reduktionsvorgänge. Die Organismen sind somit nicht nach Gram färbbar und intrinsisch resistent gegen die an der Zellwand angreifenden Antibiotika (Penizilline, Cephalosporine, Carbapeneme). Darüber hinaus sind Mykoplasmen empfindlich gegen Umwelteinflüsse und die erfolgreiche Infektion erfordert einen engen Erreger-Wirt-Kontakt. Im humanen Bereich sind inzwischen 19 *Mycoplasma*- bzw. *Ureaplasma*-Spezies beschrieben (Tab. 1). Die Bakterien zeigen eine auffällige Assoziation mit der urogenitalen bzw. respiratorischen Mukosa, werden in der überwiegenden Zahl der Infektionen nicht systemisch (kein Nachweis im Blut) und bleiben meist auf den Menschen als natürliches Reservoir beschränkt. Die medizinische Bedeutung ist für viele Arten unbekannt oder auf besondere Konstellationen beschränkt. So sind *Ureaplasma urealyticum* und *U. parvum* bei vielen Personen nachweisbar, ohne Symptome hervorzurufen. Sie sind demnach im Grenzbereich zwischen Kommensalen und Krankheitserregern anzusiedeln, die z. B. bei entsprechender Disposition (z. B. Frühgeborene) bzw. hoher Infektionsdosis in Einzelfällen als Erkrankungsursache relevant werden können. Dagegen ist der Status als Pathogene für *Mycoplasma pneumoniae* und *M. genitalium* unbestritten. Neben unterschiedlichen Übertragungswegen weisen beide Spezies aktuell besondere Aspekte in ihrer Epidemiologie auf, die eine Einordnung der gegenwärtigen Situation notwendig machen. Die vorliegende Übersicht fasst den momentanen Kenntnisstand zu beiden Erregern zusammen und zieht Schlussfolgerungen im Hinblick auf ihre Bewertung für die praktisch tätigen Medizinerinnen und Mediziner, das diagnostische Labor sowie den Öffentlichen Gesundheitsdienst. Dafür werden für beide Spezies folgende Punkte diskutiert:

- bekannte Virulenzfaktoren und Bedeutung für den Krankheitsverlauf,
- Assoziation der Infektionen durch *M. pneumoniae* und *M. genitalium *mit spezifischen Symptomausprägungen,
- Aussagekraft der verfügbaren diagnostischen Verfahren,
- Therapieempfehlungen und aktuelle Resistenzsituation in Deutschland.

**Tab. 1:** Aus dem Menschen isolierte Mykoplasmen‑/Ureaplasmenspezies

Spezies	Erstbeschreibung	Lokalisation	Pathogenität
*M. hominis*	1937	Urogenitaltrakt	+
*M. fermentans*	1952	Urogenitaltrakt?	
*U. urealyticum*/*parvum*	1954	Urogenitaltrakt	(+)
*M. salivarium*	1955	Respirationstrakt	
*M. primatum*	1955	Respirationstrakt	
*M. pneumoniae*	1962	Respirationstrakt	+++
*M. orale*	1964	Respirationstrakt	
*M. buccale*	1974	Respirationstrakt	
*M. faucium*	1974	Respirationstrakt	
*M. lipophilum*	1974	Respirationstrakt	
*M. genitalium*	1981	Urogenitaltrakt	++
*M. pirum*	1985	Urogenitaltrakt	
*M. spermatophilum*	1991	Urogenitaltrakt	
*M. penetrans*	1992	Urogenitaltrakt	
*M. amphoriforme*	2005	Respirationstrakt	(+)
*Cand. M. girerdii*	2014	Urogenitaltrakt	
*Cand. M. haemohominis*	2020	Systemisch?	
*M. phocimorsus*	2023	Gelenkentzündungen (Zoonose)	(+)

*+/(+)* wenig ausgeprägt/nur unter besonderen Bedingungen; *++* ausgeprägt in definierten Patientengruppen; *+++* ausgeprägt in der Gesamtbevölkerung

## Mycoplasma pneumoniae

### Pathogenese

Grundsätzlich weisen Mykoplasmen aufgrund ihrer begrenzten Ausstattung auch nur ein limitiertes Repertoire an Virulenzfaktoren auf. *M. pneumoniae* besitzt eine Adhärenzstruktur, die polar angeordnet ist und die Anheftung an die Zielstrukturen in der respiratorischen Schleimhaut realisiert. Darüber hinaus besitzt dieser Proteinkomplex Bedeutung für die Teilung und die Gleitbewegung der Organismen. Das Community-acquired Respiratory Distress Syndrome-(CARDS-)Toxin weist strukturelle Ähnlichkeiten mit dem Pertussis-Toxin auf und ist, zusammen mit der Freisetzung von Radikalen (H_2_O_2_, H_2_S), verantwortlich für die lokale Zell- und Gewebeschädigung, einschließlich der Reduktion der Zilienbewegung. Die Bildung eines Biofilms dürfte die Persistenz der Mykoplasmen in der Mukosa verlängern. Die Entzündungsreaktion wird maßgeblich durch die Vielzahl der oberflächenassoziierten Lipoproteine ausgelöst [1]. Darüber hinaus sind die Bakterien in der Lage, das Immunsystem des Wirtes zu beeinflussen. Dazu wird ein Immunglobulin-bindendes Protein gebildet [2]. Obwohl *M. pneumoniae* genetisch als außergewöhnlich konserviert zu charakterisieren ist, können bestimmte Gene, wie z. B. das für das Hauptadhäsin P1 kodierende Gen, darüber hinaus durch homologe Rekombination repetitiver Elemente verändert werden. Da diese oberflächenassoziierten Adhärenzproteine in vielen Fällen maßgeblich für die Immunantwort des Wirtes sind, kann der Wechsel der zirkulierenden P1-Typen die Wirksamkeit der vorherrschenden Immunität in der Population vorübergehend beeinträchtigen.

### Epidemiologie und Nachweis

Die morphologisch sehr kleinen *M.-pneumoniae*-Zellen (ca. 0,2 × 2,0 µm) werden durch Aerosole bei engem Mensch-zu-Mensch-Kontakt übertragen. Die Basisreproduktionszahl R_0_ ist mit 1,7 relativ niedrig und spricht für eine geringe Ausbreitungsdynamik des Erregers [3]. Kleinräumige Ausbrüche werden deshalb vorrangig z. B. aus Familien, Kindertagesstätten, Schulen und auch aus militärischen Ausbildungseinrichtungen berichtet. Die Infektionen sind überwiegend ambulant erworben. Für nosokomial erworbene Pneumonien spielt der Erreger nur eine untergeordnete Rolle [4]. Obwohl alle Altersgruppen betroffen sein können, zeigt sich eine typische Altersverteilung mit einer Häufung bei älteren Kindern und jungen Erwachsenen. Während die Infektion bei der überwiegenden Zahl der Patientinnen und Patienten zu einer Tracheobronchitis führt, entwickelt sich bei ca. 10 % der Infektionen eine atypische Pneumonie. Die Inkubationszeiten sind mit 1–3 Wochen (zum Teil darüber) relativ lang. Die Pneumonie ist oft mild und selbstlimitierend, jedoch werden regelmäßig schwerere Verläufe beschrieben, die in Einzelfällen eine intensivmedizinische Behandlung erforderlich machen können [5, 6]. Todesfälle stellen jedoch die absolute Ausnahme dar. In diesem Zusammenhang werden aufgrund der erwähnten Eigenschaften der Bakterien keine über die Basishygiene hinausgehenden Maßnahmen im Krankenhaus empfohlen [7]. Da die Erkrankung oft schleichend und in den meisten Fällen nicht mit hohem Fieber, Kopfschmerzen und starkem Krankheitsgefühl beginnt, wird die Infektion in den ersten Tagen unterschätzt. Erst ein sich steigernder quälender Hustenreiz sowie Antriebsschwäche, subfebrile und dann langsam ansteigende Temperaturen sind Anlass für eine Untersuchung. Die produzierte Sputummenge ist eher gering. Es fehlen bei Beteiligung des unteren Respirationstrakts die sonst für bakteriell verursachte Pneumonien typischen grobblasigen Rasselgeräusche. Bei Mykoplasmenpneumonien sind sie eher diskret, sehr feinblasig und in den unteren Quadranten zu finden. Erst das Thorax-Röntgenbild gibt Aufschluss über das Ausmaß der interstitiellen Pneumonie. Eine Besonderheit sind extrapulmonale Manifestationen, die u. a. das Zentralnervensystem (Enzephalitis), die Haut (*Mycoplasma pneumoniae* Induced Rash and Mucositis; MIRM) oder das Herz-Kreislauf-System betreffen können. Diese Komplikationen sind bei ca. 6 % der Infizierten zu diagnostizieren [1] und können Monate nach der Primärinfektion bzw. z. T. ohne Erregernachweis auftreten. Die Pathogenese ist nicht abschließend geklärt; in vielen Fällen werden Autoimmunphänomene eine Rolle spielen [8], da die Zellmembran der Mykoplasmen der Wirtszelle ähnlicher als die Oberfläche der zellwandtragenden Bakterien sein dürfte.

Obwohl die Infektionen über das ganze Jahr verteilt auftreten, war in der Vergangenheit in Europa ein Anstieg während der Herbst- und Wintermonate zu verzeichnen. Bereits seit Jahrzehnten werden darüber hinaus Zunahmen der gemeldeten Fälle in einem Zyklus von 3 bis 7 Jahren beobachtet, die zum Teil weltweit zu registrieren waren. Als Ursache wird neben dem erwähnten Wechsel der Dominanz von P1-Typen in der Population ein Abfall der spezifischen Immunität während der Zeit eines endemischen Vorkommens der Infektionen diskutiert [9].

Der Nachweis von Infektionen durch *M. pneumoniae* kann prinzipiell durch eine Kultur erfolgen. Aufgrund der langen Bebrütungszeiten (> 10 Tage) und der begrenzten Sensitivität ist dieses Verfahren für klinische Fragestellungen obsolet. Die Bestätigung im Labor erfolgt in der Regel durch die Polymerase-Kettenreaktion (PCR). Vor allem im ambulanten Bereich wird darüber hinaus der serologische Nachweis eingesetzt. Probleme mit der Sensitivität/Spezifität einzelner Testsysteme, der verzögerten Reaktion auf die Infektion, der Persistenz der spezifischen Immunglobuline oder die oft ausbleibende IgM-Antwort bei Reinfektionen im Erwachsenenalter können jedoch die Aussagekraft der Resultate beeinträchtigen. Der Nachweis von IgG-Antikörpern ist somit bei Vorliegen gepaarter Seren (Untersuchung eines Zweitserums ca. 2 Wochen nach dem Erstserum) sinnvoll. Die Detektion von spezifischem IgM kann insbesondere bei pädiatrischen Fällen eine Verdachtsdiagnose unterstützen. Darüber hinaus bereitet die Trennung von asymptomatischen Trägern und symptomatisch Infizierten sowohl für die PCR als auch den serologischen Nachweis Probleme bei der Bewertung der Ergebnisse. Die Rate *M.-pneumoniae*-positiver symptomloser Kinder liegt bei rund 20 % [10], ist höher bei wiederkehrenden Infektionen und kann bis zu 50 % der Haushaltangehörigen erkrankter Personen betreffen [11]. Der langfristige Nachweis des Erregers über Wochen und Monate trotz adäquater Therapie und klinischer Besserung ist beschrieben [12].

**Abb. 1:**
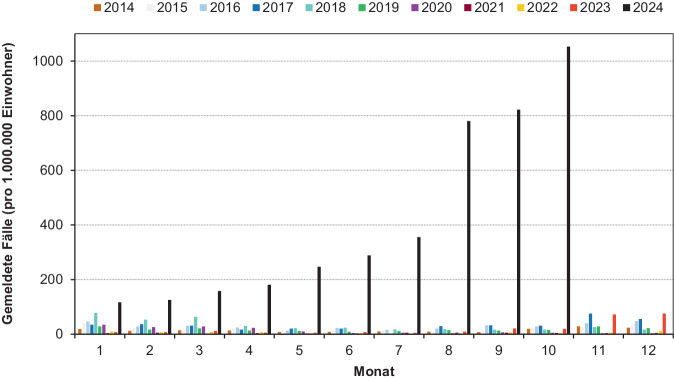
Prävalenz der gemeldeten Infektionen mit *M.* *pneumoniae* im Freistaat Sachsen, 2014–2024 (*Quelle*: Landesuntersuchungsanstalt Sachsen, https://www.lua.sachsen.de, eigene Abbildung)

Ausgehend von ersten Berichten über hohe Zahlen respiratorischer Infektionen im Jahr 2023 bei Kindern in China [13] hat sich in den vergangenen Monaten eine außergewöhnliche Inzidenz von durch *M. pneumoniae* verursachten Krankheitsfällen in vielen europäischen Ländern entwickelt [14–16]. Aufgrund der vergleichsweise begrenzten klinischen Relevanz der Infektionen durch den Erreger erfolgt eine Zählung der gemeldeten Labornachweise in Deutschland ausschließlich in Sachsen, sodass die verfügbare Datenbasis begrenzt ist. Die Meldedaten beinhalten neben indirekten (Serologie) überwiegend direkte Nachweise (PCR). Die Altersverteilung im Jahr 2024 bestätigt ein überwiegendes Vorkommen in der Altersgruppe 5–14 Jahre (52 %) und hat sich gegenüber 2023 nicht verändert. Dies gilt auch für den Anteil jüngerer Erwachsener (25–44 Jahre). Der Aufwand für die Erfassung der Fälle in der Landesuntersuchungsanstalt Sachsen gestattet zumindest ein Bild der lokalen Situation (Abb. 1). Obwohl verschiedene Faktoren die Meldestatistik beeinflussen werden (zunehmender Einsatz von Multiplex-Nachweisen, Sensibilisierung in den hausärztlichen und pädiatrischen Praxen für den Nachweis durch Berichte über die Häufung von *M.-pneumoniae*-Infektionen, Probleme des serologischen Nachweises), kann jedoch davon ausgegangen werden, dass die Lage in Sachsen grundsätzlich auf andere Bundesländer übertragbar ist. Danach ist mit Beginn im November 2023 bis Oktober 2024 eine kontinuierliche Zunahme der Meldungen festzustellen, die die Daten für die vergangenen Jahre um ein Vielfaches übersteigt. Neben der hohen Inzidenz ist die fehlende Abnahme der Meldezahlen über die Sommermonate außergewöhnlich. Zu den Ursachen dieser besonderen Entwicklung werden die 2 bereits erwähnten Hypothesen (Subtypwechsel bzw. die Abnahme der spezifischen Immunität während der COVID-19-Pandemie) diskutiert. Die Maßnahmen zur Verringerung respiratorischer Infektionen während der COVID-19-Pandemie haben national und international auch zu einer signifikanten Abnahme der Infektionen durch *M. pneumoniae* geführt [17]. Diese Entwicklung war für weitere Erreger von Atemwegsinfektionen bereits dokumentiert. Damit ist von einem verringerten Kontakt der Bevölkerung mit den Bakterien über einen längeren Zeitraum und einer Abnahme der gegen den Erreger gerichteten Immunität auszugehen. Für die postpandemische Zeit bestätigen erste Studien niedrigere Konzentrationen spezifischer Immunglobuline gegen *M. pneumoniae* im Vergleich zu vor der COVID-19-Pandemie [18]. Dagegen gibt es bisher keine Hinweise auf das Auftreten neuer oder die Dominanz einzelner Genotypen. Die im Konsiliarlabor durchgeführte Typisierung von Stämmen (*n* = 121) aus der aktuellen Hochinzidenzperiode in Deutschland zeigte, dass der Ausbruch polyklonal ist. Als Grund für die im Vergleich zu anderen respiratorischen Erregern verspätete Zunahme der Infektionen Ende 2023 kann die besondere Charakteristik der Bakterien (lange Generations- und Inkubationszeiten) angeführt werden, die offensichtlich auch ihre Anpassung an die Situation nach der Pandemie beeinflusst hat. Es ist jedoch grundsätzlich anzunehmen, dass sich mit der Infektion größerer Bevölkerungsgruppen ein den Vorjahren vergleichbarer Immunstatus einstellt, der mit der Abnahme der hohen Inzidenzen einhergehen wird. Obwohl die Datenlage nicht eindeutig ist, nimmt die Zahl schwerer Infektionen aufgrund der deutlich erhöhten Inzidenz erwartungsgemäß zu, die Rate bleibt jedoch in Europa relativ konstant [19–21].

### Therapie und Resistenz

**Abb. 2:**
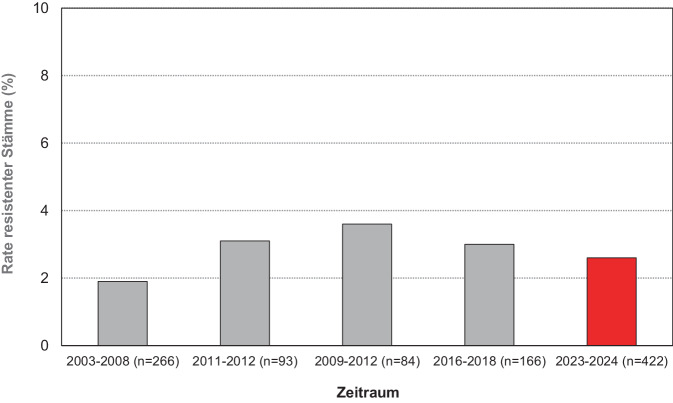
Makrolidresistenzraten von *M.-pneumoniae*-Stämmen aus Deutschland (Ergebnisse unabhängiger Studien im Konsiliarlabor, eigene Abbildung)

Grundsätzlich bedarf nicht jede nachgewiesene Infektion mit *M. pneumoniae* einer antibiotischen Behandlung. Entsprechend der aktuellen Leitlinie [22] kann bei sorgfältiger klinischer Untersuchung und Beobachtung der Betroffenen aufgrund der meist milden Verläufe zunächst abgewartet werden. Bei schwereren Krankheitsbildern ist eine Therapie angezeigt. Grundsätzlich sind neben Makroliden auch Tetrazykline und Chinolone wirksam, wobei Letztere in der Gruppe der häufig betroffenen Kinder in der Anwendung eingeschränkt bzw. kontraindiziert sind. Entsprechend den nationalen Empfehlungen bleiben Makrolide in der pädiatrischen Praxis die Antibiotika der Wahl, während für Erwachsene auch Doxycyclin empfohlen wird [22, 23]. In Abhängigkeit von der Schwere des Krankheitsbildes ist mindestens 5 Tage antibiotisch zu behandeln. Zukünftige Studien müssen zeigen, ob eine kürzere Therapiedauer sinnvoll sein kann. Die Resistenzlage in Deutschland ist günstig. Es sind national und international bisher keine klinischen Isolate mit Tetrazyklin- oder Chinolonresistenz nachgewiesen worden, obwohl diese durch eine *In-vitro*-Passagierung empfindlicher Stämme in Anwesenheit subinhibitorischer Antibiotikakonzentrationen induziert werden kann [24]. Im Gegensatz zur Situation in China (Resistenzraten um 90 %) sind die in Deutschland zirkulierenden Stämme überwiegend sensibel gegenüber den gut verträglichen Makroliden. Obwohl Unterschiede in der Empfindlichkeit von Stämmen bestehen, die Mutationen in der Binderegion der 23S rRNA von *M. pneumoniae* aufweisen, ist für die häufig nachgewiesenen Veränderungen mit minimalen inhibitorischen Konzentrationen > 16 µg/ml für Azithromycin und Clarithromycin zu rechnen [25]. Die Rate resistenter Stämme wird seit mehr als 20 Jahren im Konsiliarlabor verfolgt und hat sich in diesem Zeitraum nur wenig verändert (Abb. 2). Bei Fortführung der Verschreibungspraxis gibt es gegenwärtig keinen Anlass, mit einer Zunahme der Resistenzrate gegenüber Makroliden zu rechnen, die mit der Situation bei *M. genitalium* vergleichbar ist (siehe unten). Die aktuellen Zahlen belegen damit auch, dass ein verändertes Resistenzmuster als Ursache der hohen Inzidenzen keine Rolle spielt. Es sei jedoch betont, dass Resistenzentwicklungen während der Makrolidtherapie bestätigt wurden [26, 27] und fehlendes klinisches Ansprechen (persistierender Husten, kein Fieberrückgang, ggf. unveränderte respiratorische Situation bei hospitalisierten Patientinnen und Patienten) Anlass für die Untersuchung des Stammes in einem Speziallabor sein sollte. Die einer Resistenz zugrunde liegenden Mutationen in der Zielstruktur der Antibiotikagruppe (23S rRNA) sind ausreichend charakterisiert. Weitere Resistenzmechanismen sind nicht bekannt. In klinischen Stämmen dominieren Mutationen an den Positionen 2058/2059 (*Escherichia-coli*-Nummerierung) der Domäne V der 23S rRNA und bedingen eine ausgeprägte Resistenz gegenüber der gesamten Substanzklasse (keine „intermediär“ empfindlichen Isolate). In der entsprechenden Richtlinie des Clinical and Laboratory Standards Institute (CLSI; [28]) sind für Tetrazyklin, Moxifloxacin/Levofloxacin und Erythromycin minimale inhibitorische Konzentrationen festgelegt (z. B. für eine Erythromycinresistenz: ≥ 16,0 µg/ml), sodass das Fehlen von Isolaten für die phänotypische Resistenzbewertung dieser Spezies bisher praktisch kein Problem darstellt. Kommerzielle Verfahren für die molekulare Testung des Vorliegens einer Makrolidresistenz sind verfügbar und validiert [29]. Der Verlauf der Infektionen durch Makrolid-resistente Stämme ist durch eine Verlängerung der Fieber- und Erkrankungszeit und ggf. der Hospitalisierungsphase gekennzeichnet. Insbesondere in Regionen mit hohen Resistenzraten sind jedoch auch eine Verstärkung der Symptomatik im Sinne einer refraktären Pneumonie und eine erhöhte Rate extrapulmonaler Manifestationen beschrieben [30].

## Mycoplasma genitalium

### Pathogenese und Nachweis

*M. genitalium* ist genetisch eng mit *M. pneumoniae* verwandt [31]. Auch bei diesem Erreger sind eine Vielzahl von Lipoproteinen und ein Attachment-Organell zu finden, das ebenfalls Komponenten mit variablen Bereichen aufweist. Ein dem CARDS-Toxin vergleichbares Protein ist bei *M. genitalium* jedoch nicht beschrieben [32].

Für den Nachweis der Mikroorganismen wird im diagnostischen Labor ausschließlich die PCR eingesetzt. Es entwickelt sich eine spezifische Immunantwort nach der Infektion, validierte serologische Tests werden jedoch bisher nicht angeboten. Darüber hinaus ist eine Kultur prinzipiell möglich, dauert jedoch Wochen und Monate und wird nur von wenigen Referenzlaboren beherrscht. Die geringe Zahl verfügbarer Isolate ist insbesondere für die Bewertung der klinischen Relevanz bestimmter Mutationen als kritisch einzuschätzen [33].

### Epidemiologie

*M. genitalium* ist ein sexuell übertragbarer Erreger, der abseits von bestimmten Risikogruppen (z. B. Sexarbeitende oder Männer, die Sex mit Männern haben; MSM) bei etwa 1–4 % der Personen nachweisbar ist. Innerhalb von Risikogruppen kann diese Rate auf über 20 % ansteigen [34]. Primäre Lokalisation ist der Urogenitaltrakt; Rektum und Pharynx können ebenfalls kolonisiert sein [35]. Unbestritten ist die Rolle des Erregers als Urethritisursache bei Männern, bei denen andere Mikroorganismen, die eine entsprechende Symptomatik auslösen können (Gonokokken, Chlamydien), nicht nachgewiesen werden. In bis zu 40 % dieser Fälle führt die auf Mykoplasmen ausgerichtete Behandlung zu einer signifikanten Besserung der Symptomatik. Darüber hinaus wird *M. genitalium* im Zusammenhang mit Epididymitis, Zervizitis, der Pelvic Inflammatory Disease, Infertilität und Frühgeburtlichkeit diskutiert [36]. Dennoch bleibt die Infektion bei vielen Patientinnen und Patienten asymptomatisch [37] und der Wirt kann den Erreger ohne Behandlung verlieren [38]. *M. genitalium* ist als Risikofaktor für eine HIV-Infektion anzusehen, wobei sich die bisherigen Erkenntnisse vorwiegend auf Frauen konzentrieren. Eine Metaanalyse bestätigt die Assoziation von *M.-genitalium*- und HIV-Infektion [39]. Mit zunehmender Mykoplasmenbesiedlung steigt die Wahrscheinlichkeit der Ausscheidung höherer Konzentrationen an HI-Viren.

### Therapie und Resistenz

Ein Screening asymptomatischer Patientinnen und Patienten auf *M. genitalium* ist grundsätzlich abzulehnen. Neben den erwähnten Urethritisfällen bei Männern, bei denen ursächlich an *M. genitalium* gedacht werden sollte, ist der Erreger jedoch in viele der zunehmend eingesetzten Multiplex-Nachweise für sexuell übertragene Infektionen integriert. Die dann meist „ungezielte“ Detektion von *M. genitalium* führt insbesondere bei asymptomatischen Personen zu Unsicherheiten hinsichtlich der Notwendigkeit einer Therapie. Zur Vermeidung weiterer Infektionen wäre eine Behandlung bei sexuell aktiven Personen mit Partnerwechsel und eingeschränktem Verhütungsverhalten aus infektiologischer Sicht jedoch durchaus wünschenswert. Prinzipiell werden nach europäischer und weiteren Leitlinien Makrolide (Azithromycin, Erstrangantibiotikum) gefolgt von Fluorchinolonen (Moxifloxacin) empfohlen. Doxycyclin kann eingesetzt werden, zeigt aber nur eine klinische Wirksamkeit von etwa 35 % [37]. Bei festen sexuellen Beziehungen ist eine Partnerbehandlung angezeigt. Aufgrund der nachfolgend beschriebenen Resistenzsituation ist die Untersuchung einer Follow-up-Probe sinnvoll (frühestens 3 Wochen nach dem Ende der Antibiose).

**Abb. 3:**
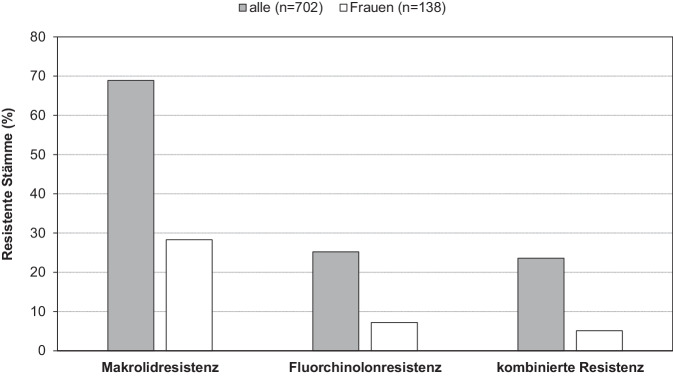
Resistenzraten von an das Konsiliarlabor für Mykoplasmen des Robert Koch-Instituts eingesandten *M.-genitalium*-positiven Materialien, 2021–2024, eigene Abbildung

Da die epidemiologische Bedeutung von *M. genitalium* nach bisherigen Erkenntnissen als begrenzt anzusehen ist, führte vor allem die Resistenzentwicklung in den vergangenen Jahren zu verstärkten Diskussionen um den Erreger. In dieser Hinsicht weisen die beiden hier diskutierten Spezies signifikante Unterschiede in der Empfindlichkeit gegenüber den für die Therapie empfohlenen Antibiotikaklassen auf. Wie bei *M. pneumoniae* gilt auch für *M. genitalium*, dass ausschließlich Mutationen der Zielstrukturen der Antibiotika als Mechanismen für eine Resistenzentwicklung nachgewiesen wurden. So sind ebenfalls vor allem 2 Mutationen in der 23S rRNA zu nennen (2058/2059, *E.-coli*-Nummerierung), die zu einer Makrolidresistenz des Stammes führen. Im Gegensatz zu *M. pneumoniae* werden bei *M. genitalium* jedoch zunehmend Mutationen des Zielproteins (Topoisomerase ParC) für die Fluorchinolonwirkung festgestellt. Aufgrund des Fehlens entsprechender Isolate für die phänotypische Empfindlichkeitstestung bleibt jedoch die Feststellung des Zusammenhangs zwischen Mutation und Therapieversagen oft auf die klinische Beobachtung beschränkt. Inzwischen sind mehrere Aminosäurenaustausche in der Quinolone-Binding-Region des ParC-Proteins in Stämmen beschrieben. Es wird jedoch gegenwärtig davon ausgegangen, dass vorrangig die Mutationen S48 bzw. D59 zur Resistenz führen. Sie sind in vielen Studien auch die am häufigsten nachgewiesenen Veränderungen. Solitäre Mutationen der Gyrase A führen nach aktuellem Stand nicht zu Fluorchinolonresistenz. In Kombination mit *parC*-Mutationen sind jedoch Erhöhungen der minimalen inhibitorischen Konzentrationen zu erwarten [40]. Die Resistenzsituation kann lokal unterschiedlich ausgeprägt sein. Für die genannten Risikogruppen mit einer meist umfangreichen Historie von Antibiotikabehandlungen ist jedoch von Raten über 50 % (Makrolide) bzw. über 10 % (Fluorchinolone) auszugehen [41]. Zur Untersuchung gehen im Konsiliarlabor für Mykoplasmen überwiegend (ca. 90 %) *M.-genitalium*-positive Proben ein, die vor der Therapie getestet werden. Die Einsendung von Stämmen nach Therapieversagen ist nicht grundsätzlich auszuschließen, es ist jedoch nicht von einem erheblichen Einfluss dieser Selektion auf die gewonnenen Resultate auszugehen. Die Auswertung der Daten aus den vergangenen Jahren (*n* = 702) ergab für Deutschland eine Makrolidresistenz in 68,9 % und eine Fluorchinolonresistenz in 25,2 % aller Fälle (Abb. 3). Da bei der überwiegenden Zahl der Einsendungen keine Informationen zur sexuellen Orientierung bzw. zur Einordnung in Risikopopulationen vorliegen, wurden Patientinnen gesondert betrachtet. Auch in dieser Gruppe (*n* = 138) ist die Resistenz der Stämme zwar signifikant geringer, aber inzwischen ebenfalls besorgniserregend (Makrolide: 28,3 %, Fluorchinolone: 7,2 %). In Risikogruppen ist mit einem höheren Anteil resistenter Stämme zu rechnen [42]. Eine Fluorchinolonresistenz geht in der überwiegenden Zahl der Fälle (94 %) mit einer Makrolidresistenz einher. Insbesondere die Behandlung von Infektionen mit mehrfach resistenten Stämmen ist schwierig. Nach europäischer Leitlinie [37] kann bei Vorliegen von Makrolid- und Fluorchinolonresistenz eine Therapie mit den in Deutschland nicht zugelassenen Präparaten Pristinamycin (Bezug über die Internationale Apotheke, momentan ausgeprägte Lieferprobleme) und Minocyclin vorgenommen werden. Die zu erwartende Erfolgsrate liegt bei 70–75 % [35]. Einzelversuche einer erfolgreichen Behandlung mit weiteren Alternativen (z. B. Chloramphenicol [43]) sind beschrieben.

Kommerzielle Verfahren vorrangig zum Nachweis der Makrolid-, aber auch einer Fluorchinolonresistenz sind in den vergangenen Jahren eingeführt worden [44]. Die fehlende Möglichkeit einer Abrechnung der Untersuchung verhinderte bisher einen breiteren Einsatz in den diagnostischen Labors. Die aktuelle Resistenzlage unterstreicht jedoch, dass eine leitlinienorientierte empirische Therapie ohne Resistenztestung bei der überwiegenden Zahl der Infizierten in Risikogruppen kaum mehr möglich ist. Dies gilt in abgeschwächter Form inzwischen auch in der heterosexuellen Population. Aus den Resistenzentwicklungen in den vergangenen Jahren, der zunehmenden Einbeziehung von Nachrang-Antibiotika in die Therapie, der begrenzten Verfügbarkeit neuer Therapeutika und dem Fehlen einer umfangreichen Resistenztestung in vielen Ländern resultierte die Befürchtung, dass Infektionen mit diesen primär relativ unkomplizierten Bakterien in naher Zukunft kaum noch behandelbar sind [45]. Mit der Aufnahme von *M. genitalium* in die Watchlist der US-amerikanischen Centers for Disease Control and Prevention (CDC) zu den Gefahren antibiotischer Resistenz wird die diesbezügliche Herausforderung deutlich [46]. Aufgrund der gegenwärtigen Empfindlichkeiten der zirkulierenden Stämme hat bei der überwiegenden Zahl der Infektionen nur eine resistenzgeleitete Therapie Aussicht auf Erfolg, mit der in über 90 % der Fälle eine Erregerelimination erreicht werden kann [47]. Entsprechende Ansätze sind z. B. in der aktuellen australischen Leitlinie verankert, die darüber hinaus eine Doxycyclintherapie zur Reduktion der Erregerlast bis zum Vorliegen der Ergebnisse der Resistenztestung empfiehlt [48]. Bei fehlender Resistenztestung rät auch die Weltgesundheitsorganisation (WHO) in Regionen mit hohen Makrolidresistenzraten zu einer Vorbehandlung mit Doxycyclin gefolgt von Moxifloxacin [49].

## Fazit

Die humanpathogenen Mykoplasmenspezies *M. pneumoniae* und *M. genitalium* sind Vertreter einer Bakteriengruppe mit besonderen Eigenschaften, die den Nachweis, die Therapie und die epidemiologische Bewertung der Infektionen maßgeblich beeinflussen. Die zeitweise außergewöhnlich hohen Inzidenzen (*M. pneumoniae*) bzw. die problematische Resistenzsituation (*M. genitalium*) stellen unterschiedliche Herausforderungen für die Praxis dar. Während für ambulant erworbene Atemwegsinfektionen Diagnostik und Therapie an die Wahrscheinlichkeit anzupassen sind, dass *M. pneumoniae* in vielen Fällen als Auslöser anzusehen ist, erfordert der zunehmende Nachweis von *M. genitalium* in Multiplex-Verfahren für die Diagnostik von sexuell übertragbaren Infektionen die Erarbeitung einer grundsätzlichen Strategie für den Umgang mit den in vielen Fällen asymptomatisch Infizierten. Die Therapieoptionen für schwerere *M.-pneumoniae*-Fälle sind in Deutschland günstig. Dagegen ist bei der Behandlung von Infektionen mit *M. genitalium* den ausgeprägten Resistenzen gegenüber den beiden vorrangig empfohlenen Antibiotika Rechnung zu tragen, dem nur durch ein resistenzgeleitetes Therapiekonzept begegnet werden kann.
